# Phosphatases: The New Brakes for Cancer Development?

**DOI:** 10.1155/2012/659649

**Published:** 2011-10-31

**Authors:** Qingxiu Zhang, Francois X. Claret

**Affiliations:** Department of Systems Biology, The University of Texas MD Anderson Cancer Center, 1515 Holcombe Boulevard, Houston, TX 77030, USA

## Abstract

The phosphatidylinositol 3-kinase (PI3K) pathway plays a pivotal role in the maintenance of processes such as cell growth, proliferation, survival, and metabolism in all cells and tissues. Dysregulation of the PI3K/Akt signaling pathway occurs in patients with many cancers and other disorders. This aberrant activation of PI3K/Akt pathway is primarily caused by loss of function of all negative controllers known as inositol polyphosphate phosphatases and phosphoprotein phosphatases. Recent studies provided evidence of distinct functions of the four main phosphatases—phosphatase and tensin homologue deleted on chromosome 10 (PTEN), Src homology 2-containing inositol 5′-phosphatase (SHIP), inositol polyphosphate 4-phosphatase type II (INPP4B), and protein phosphatase 2A (PP2A)—in different tissues with respect to regulation of cancer development. We will review the structures and functions of PTEN, SHIP, INPP4B, and PP2A phosphatases in suppressing cancer progression and their deregulation in cancer and highlight recent advances in our understanding of the PI3K/Akt signaling axis.

## 1. Introduction

The phosphatidylinositol (PI) 3-kinase (PI3K) signaling pathway is a normal signal transduction cascade that exists in all types of cells and is physiologically involved in cell proliferation, survival, protein synthesis, metabolism, differentiation, and motility. In physiological situations, many growth factors and regulators can stimulate or activate this pathway. The PI3K pathway contains the upstream PI3K, which phosphorylates the D-3 position of PI, PI 4-phosphate, and PI 4,5-bisphosphate (PIP_2_) to produce PI 3-phosphate, PI 3,4-bisphosphate (PI(3,4)P_2_), and PI 3,4,5-trisphosphate (PI(3,4,5)P3 or PIP_3_), respectively [1], as well as Akt and its kinases PDK1, targets at Thr308 of Akt, and PDK2 which targets at Ser473 of Akt [2]. The second messengers of PIs are associated with major cellular functions such as growth, differentiation, apoptosis, protein trafficking, and motility. Several studies have identified inositol polyphosphate phosphatases, including three major PIP2/PIP_3_-degrading enzymes: (1) phosphatase and tensin homologue deleted on chromosome 10 (PTEN), an ubiquitously expressed tumor suppressor that converts PI(3,4,5)P_3_ to PI(4,5)P_2_ by dephosphorylating the 3-position of PI(3,4,5)P3; (2) Src homology 2 (SH2)-containing inositol 5′-phosphatase (SHIP), which dephosphorylates the 5-position PI(4,5)P3 to produce PI(4)P and hydrolyzes PI(3,4,5)P3 to PI(3,4)P_2_ phosphatase [3]; (3) inositol polyphosphate 4-phosphatase type II (INPP4B), which hydrolyzes the 4-position phosphates of PI(3,4)P_2_ [4, 5] and LKB1 [6] of the downstream tuberous sclerosis complex 2 (TSC2) [7, 8] and eukaryotic initiation factor 4E-(eIF4E) [9–11]. Besides these three major lipid phosphatases, other phosphatases inhibit the PI3K/Akt pathway, such as the serine/threonine phosphoprotein phosphatase (PPP) family member PP2A [12, 13]. The PPP family has seven members: PP1, PP2A, PP2B (commonly known as calcineurin), PP4, PP5, PP6, and PP7. PP1 and PP2 are the most abundant and ubiquitous serine/threonine protein phosphatases in this family. To date, PP2A is the only known Akt-Thr308 phosphatase [14, 15]. Unlike PP1 and PP2A, the in vitro basal activity of PP4, PP5, PP6, and PP7 is extremely low. PP2C (pleckstrin homology domain leucine-rich repeat protein phosphatase) belongs to a novel PP2C-type phosphatase family, the PPM subfamily. Pleckstrin homology domain leucine-rich repeat protein phosphatase functions as a “brake” for Akt and protein kinase C signaling, which has been extensively reviewed [16]. Herein we describe the structures of PTEN, SHIP, INPP4B, and PP2A phosphatases. We also characterize their functions in tumorigenesis and highlight our current knowledge of the PI3K/Akt pathway.

## 2. PTEN

### 2.1. PTEN Function: The Main Brake for Tumor Development

*PTEN*/*MMAC* (mutated in multiple advanced cancers), that controls negatively the PI3K/Akt pathway, is a tumor suppressor gene. PTEN normally inhibits PI3K/AKT activation by dephosphorylating PIP_3_ and PIP_2_, thus suppressing tumor formation [3, 17–19]. Two groups initially and simultaneously identified *PTEN*/*MMAC *as a candidate tumor suppressor gene located at 10q23 [20, 21]. Another group found that the protein transforming growth factor-(TGF-)*β*—regulated and epithelial cell-enriched phosphatase 1 encoded by the *TEP1* gene is identical to the protein encoded by the candidate tumor suppressor gene PTEN/MMAC1 in a search for new dual-specificity phosphatases [22]. Loss of heterozygosity of PTEN at chromosome 10q22–25 occurs in multiple tumor types, most prominently advanced glial tumors (glioblastoma multiforme and anaplastic astrocytoma) but also prostate, endometrial, renal, and small cell lung carcinoma; melanoma; meningioma. Germline mutations in *PTEN* are present in cases of *Cowden *disease and *Bannayan-Zonana* syndrome, two related hereditary cancer-predisposition syndromes associated with elevated risk of breast and thyroid cancer [23, 24]. Somatic mutations and biallelic inactivation of PTEN are frequently observed in high-grade glioblastomas, melanomas, and cancers of the prostate and endometrium, among others [25]. 

Loss of PTEN function leads to increased concentrations of PIP_3_, the main in vivo substrate of PTEN, resulting in constitutive activation of downstream components of the PI3K pathway, including the kinases AKT and mammalian target of rapamycin, mTOR [3]. One study found that 37 (36%) of 103 endometrial cancers exhibited PTEN-negative immunohistochemical staining and a significant inverse correlation between expression of PTEN and that of phosphorylated AKT [26]. Another study has observed PTEN loss in both late- and early-stage melanoma cases [27]. In addition, an in vivo loss-of-function assay showed that Pten^+/−^ mice experienced spontaneous development of tumors of various histological origins [17, 18]. Moreover, PTEN inactivation dramatically enhanced the ability of embryonic stem cells to generate tumors in nude and syngeneic mice. An early study found only 2% of *PTEN *mutations in hormone receptor-positive breast cancers and identified about 20% of all *PTEN* mutations in breast cancer cell lines [28]. This suggested that *PTEN *mutation-associated cell lines are more viable in culture than patient tumors. Recent studies have shown that the frequencies of breast cancer cases associated with a loss of PTEN expression are, respectively, 30% in primary tumors and 25% in metastatic tumors [29], both higher values than those reported earlier by Stemke-Hale et al. [28]. Thus, regulation of PTEN expression at the posttranscriptional level plays a more critical role in breast cancer development compared to any genomic variations in PTEN. Besides breast cancers, researchers have characterized about 38% of patients with nonsmall cell lung cancer as having *PTEN* deletions/mutations [30]. Interestingly, Forgacs and colleagues have reported a relatively low frequency (<10%) of somatic intragenic *PTEN* mutations in small-cell lung cancers and only two silent mutations and two apparent homozygous deletions in 22 primary small-cell lung cancer tumors and metastases [31]. Also, loss of heterozygosity of the *PTEN*/*MMAC1* locus has been found in all histologic types of primary lung cancer [31]. More than 33% of *PTEN *allelic deletions occurred before lung metastasis developed [32]. In prostate cancer, the rates of *PTEN *loss of heterozygosity have been much higher. Specifically, about 56% of prostate tumors have heterozygous alterations in PTEN at presentation, and about 90% of metastases have loss of the same allele [33].

In summary, PTEN performs differently in suppressing cancer progression in various tissues because of inconsistent occurrence of loss-of-function mutations.

## 3. INPP4B

INPP4B was initially isolated from rat brain and shown to be an enzyme that primarily hydrolyzes the 4-position phosphate of PI(3,4)P_2_ into PI(3)P in vivo and slightly hydrolyzes PI(3,4,5)P_3_ in vitro [34, 35].

### 3.1. INPP4B Structure

Although the INPP4A*α* and INPP4B*α* isoforms have hydrophilic C-terminus regions, the INPP4A*β* and INPP4B*β* isoforms have hydrophobic C-termini that contain potential transmembrane domains. Additionally, INPP4A and INPP4B share 37% amino acid identity. The murine *Inpp4b* locus was mapped on chromosome 8 in a synthetic synthesized region of the human 4q27–31 interval between *Il-15* and *Usp38*. The murine INPP4B proteins include the *α* and *β* isoforms encoded by this locus. These two isoforms contain 927 and 941 amino acids, respectively, with consensus phosphatase catalytic sites and conserved C2 domains that are highly similar to those of the human and rat homologues. The C2 domain at the N-terminus of INPP4B is the lipid-binding domain. The Nervy homology 2 domain is the internal domain as well as a C-terminal phosphatase domain. Human and murine INPP4B C2 lipid-binding domains share greater than 91% sequence identity [36]. The murine INPP4B-*α* and -*β* spliced isoforms are highly conserved and have different expression patterns and cell localization [36].

### 3.2. INPP4B and Cancer

Increasing evidence has confirmed that *INPP4B* is a tumor suppressor gene. Westbrook and colleagues identified *INPP4B *as a tumorigenesis-restraining gene in a nonbiased RNA interference-based screen for genes with functional relevance to tumor initiation and development that suppress transformation of human mammary epithelial cells [37]. INPP4B expression was silenced in malignant proerythroblasts; these cells displayed increased levels of phosphorylated Akt expression that could be reduced by reexpression of INPP4B [38]. The INPP4B locus is located on chromosome 4q31.21, a region that is frequently deleted in breast cancer cell lines and high-grade basal-like breast tumors as determined using high-resolution comparative genomic hybridization analysis [39–41]. These findings have been further supported by subsequent studies. Loss of heterozygosity of INPP4B is frequently observed in BRCA1-mutant and hormone receptor-negative breast cancer cells. Loss of INPP4B protein expression in breast and ovarian cancer cells is associated with decreased patient survival rates. In human mammary epithelial cells and breast cancer cell lines, INPP4B was able to suppress both basal [5] and insulin-like growth factor-induced Akt phosphorylation [4]. Further evidence of INPP4B as a tumor suppressor gene comes from a nonbiased RNAi-based genetic screen. The loss of INPP4B promotes the anchorage-independent growth of human mammary epithelial cells [37]. In particular, INPP4B protein expression is lost in 84% of human basal-like breast carcinomas, which are generally highly aggressive with poor clinical outcomes and frequently associated with *BRCA1* gene mutations [42]. Authors have reported that INPP4B is expressed in nonproliferative estrogen receptor-(ER-)positive normal breast cells and breast cancer cell lines but not in ER-negative breast cancer cell lines [4]. Furthermore, INPP4B knockdown in ER-positive breast cancer cells increased Akt activation, cell proliferation, and xenograft tumor growth. Conversely, reexpression of INPP4B in ER-negative, INPP4B^−/−^ human breast cancer cells reduced Akt activation and anchorage-independent growth [4]. In the same study, INPP4B protein expression was frequently lost in primary human breast carcinoma cells, associated with high clinical grade and large tumors and loss of expression of hormone receptors, and lost most often in aggressive basal-like breast carcinomas [4]. INPP4B protein expression was also frequently lost in PTEN-null tumors [5]. Androgen-ablation therapies in the treatment of advanced prostate cancers are associated with increased Akt signaling [43]. Androgens, therefore, play an important role in control of the proliferation of prostate epithelial cells, through the downregulation of Akt signaling. Activated-Akt signaling stimulates cellular proliferation, cell survival, cell cycle progression, growth, migration, and angiogenesis [44]. The expression of INPP4B, which dephosphorylates PI(3,4)P_2_ and inactivates Akt and inhibits cellular proliferation, was substantially lower in primary prostate tumors than that in normal prostate tissue [45, 46]. Levels of INPP4B are found to be induced by the androgen receptor in prostate cancer cells and play an important role in androgen-ablation therapy of prostate cancers [45]. The INPP4B expression levels should be taken in consideration when Androgen-ablation therapies are utilized for patients with advanced prostate cancers.

## 4. SHIP

The cDNA-encoding isoform of the 145-kD protein SHIP (also called SHIP1) was initially cloned from a murine hematopoietic cell line and named B6SUtAI. B6SUtAI was then identified as the novel SH2-containing inositol polyphosphate 5-phosphatase SHIP. SHIP specifically hydrolyzes PIP_3_ and inositol 1,3,4,5-tetraphosphate [47]. The same group identified and cloned human SHIP and mapped it to the long arm of chromosome 2 at the border between 2q36 and 2q37 [48].

### 4.1. SHIP1 Structure and Function

SHIP1 contains 1190 amino acids and several identifiable motifs important for protein-protein interactions, including an N-terminal SH2 domain, a central 5′-phosphoinositol phosphatase domain, two phosphotyrosine binding consensus sequences, and a proline-rich region at the carboxyl tail. Human SHIP shares 87.2% sequence identity with mSHIP [48]. 

SHIP is expressed ubiquitously in differentiated cells in the hematopoietic system [47, 49, 50], endothelial cells [51], hematopoietic stem cells, and embryonic stem cells [52]. Particularly, SHIP1 can be phosphorylated at the tyrosine of the first NPXY motif located in the N-terminal SH2 domain [53, 54] in response to activation of hematopoietic cell surface receptors, such as erythropoietin, steel factor, interleukin-3 [55, 56], interleukin-2, granulocyte-macrophage colony-stimulating factor, and macrophage colony-stimulating factor, by numerous cytokines [57]. In one study, the number of granulocyte-macrophage progenitors in both the bone marrow and spleen increased in SHIP1-knockout mice [58, 59]. SHIP1 is essential for normal bone homeostasis, as absence of SHIP1 results in severe osteoporosis [60]. SHIP1 also reduces the proliferation of osteoclasts via Akt-dependent alterations in D-type cyclins and p27 [61].

SHIP1 is an antagonist of cell growth and proliferation in the hematopoietic system. Investigators first verified SHIP1 as a tumor suppressor in conditional B-cell PTEN/SHIP1 knockout mice. They established B-cell-specific deletion of both *Pten* and *Ship* (bPTEN/SHIP^−/−^) by mating bPTEN^−/−^ mice with a novel strain of mice lacking SHIP only in B cells (bSHIP^−/−^). The mice lacking expression of PTEN and SHIP in B cells develop lethal B-cell lymphomas with similarities to human mature B-cell lymphomas. Loss of both PTEN and SHIP expression in B cells results in an aggressive, often fatal B-cell lymphoma disease. All B-cell PTEN/SHIP1-knockout mice died by 1 year of age [62], thus, suggesting that SHIP1 and PTEN coordinately suppress B lymphoma development.

### 4.2. SHIP2 Structure and Function

The SHIP isozyme SHIP2, also named INPPL1, is a 155-kD phosphatase that is more widely expressed than is SHIP1 [63]. The SHIP2 cDNA was initially cloned from skeletal muscle, and the lipid phosphatase that hydrolyzes the 5′-phosphate of the inositol ring from in PIP_3_ was identified. SHIP2 is more broadly detected than SHIP1, which is mainly expressed in hematopoietic cells [64]. Human SHIP2 is highly expressed in adult heart, skeletal muscle, and placenta. SHIP2 regulates insulin signaling, and genetic SHIP2 knockout prevents diet-induced obesity in mice [65]. SHIP2 also regulates cytoskeleton remodeling and receptor endocytosis. In another study, SHIP2 expression was elevated in 44% of clinical breast tumor specimens [66]. Furthermore, SHIP2 is a positive regulator of the epidermal growth factor receptor/Akt pathway, C-X-C chemokine receptor type 4 expression, and cell migration in MDA-MB-231 breast cancer cells [67].

Despite the potential microRNAs (miRNAs) to regulate approximately one third of the entire genome, relatively few miRNA targets SHIP2 have been validated experimentally, particularly in stratified squamous epithelia. Yu and colleagues showed that miRNA-205 suppresses the expression of lipid phosphatase SHIP2 in epithelial cells [68]. They found that SHIP2 levels correlate reciprocally with elevated miRNA-205 levels in aggressive squamous cell carcinoma (SCC) cells. Downregulation of miRNA-205 expression in squamous cell carcinoma cells leads to decreased phosphorylated Akt and phosphorylated Bcl-2—associated death promoter expression and increased apoptosis [68]. The function of miRNA-205 in SHIP2 expression is negatively regulated by miRNA-184 in keratinocytes. Downregulation of miRNA-205 expression by ectopic expression of miRNA-184 increases SHIP2 expression and impairs the ability of keratinocytes wound healing. Keratinocytes not only express the epidermal growth factor (EGF) receptor but also produce ligands for this receptor, including TGF-*α*, amphiregulin, and HB-EGF. EGF and TGF-*α* promote keratinocyte proliferation and migration [69]. Many cellular processes, such as altered cell adhesion, expression of matrix-degrading proteinases, and cell migration, are common to keratinocytes during wound healing and in metastatic tumors. Yu and colleagues provided abundant evidence that SHIP2 is involved in keratinocyte migration promoted by miRNA-205 [70].

## 5. PP2A

PP2A is a major serine/threonine protein phosphatase in mammalian cells. It accounts for up to 1% of all cellular proteins and, together with PP1, accounts for 90% of all serine/threonine phosphatase activity in most tissues and cells [71]. PP2A is highly conserved from yeast to humans, and its regulatory mechanism is extraordinarily complex.

### 5.1. PP2A Structure and Function

Several holoenzyme complexes of PP2A have been isolated from a variety of tissues and extensively characterized. The core enzyme of PP2A is a dimer (PP2AD) consisting of a 65-kD scaffolding A subunit (also termed PR65/A and PP2R) and a 36-kD catalytic C or A subunit. The scaffolding A*α* subunit of PP2A contains 15 Huntington, elongation factor 3, a subunit of PP2A, and target of rapamycin 1 repeats [72]. The third regulatory B subunit of PP2A, which includes at least 18 regulatory subunits that have been classified B (B55 or PR55), B′ (B56 or PR61), B′′ (PR48/PR72/PR130), and B′′′ (PR93/PR110), is associated with the core enzyme. Studies identified a unique C-terminal tail (residues 294–309) in PP2A's C subunit, which contains a motif (TPDY307FL309) that is highly conserved and exists in the catalytic subunits of all PP2A-like phosphatases, including PP4 and PP6. Methylation of Leu309 in this C-terminal tail can promote recruitment of the regulatory B/B′/B′′ subunits to the A/C dimer [73]. The Huntington, elongation factor 3, a subunit of PP2A, and target of rapamycin 1 repeats in the scaffold A subunit play roles in holding the catalytic C and regulatory B′ subunits together. To date, researchers have identified five primary members of the B56 family (*α*, *β*, *γ*, *δ*, and *ε*) that are encoded by different genes—PPP2R5A, PPP2R5B, PPP2R5C, PPP2R5D, and PPP2R5E—which are mapped to the loci 1q41, 11q12, 3p21, 6p21.1, and 7p11.2, respectively [74]. B56 subunits of PP2A share a highly conserved central region of 80% identity (which comprises two A-subunit binding domains). These regulatory B subunits play key roles in controlling PP2A substrate specificity, cellular localization, and enzymatic activity [75]. These regulatory subunits are expressed in specific tissues and lead to the formation of different PP2A complexes mammalian tissues [76]. In comparison, three subunits of B56 family—B56*β*, B56*δ*, and B56*ε*—exist primarily in the brain, whereas two others—B56alpha and B56gamma—are highly expressed in cardiac and skeletal tissue [74]. PP2A expression is regulated by both C-terminal methylation and phosphorylation of the C subunit residue Tyr307; tyrosine kinases such as Src inhibit PP2A activity [77], and phosphorylation of the B56 subunit by Erk inhibits PP2A assembly [78]. 

The active core dimer of PP2A interacts with a wide variety of regulatory subunits (B subunits) and generates more than 60 different heterotrimeric PP2A holoenzymes that dictate the functions of individual forms. These regulatory subunits typically increase the formation of stable complexes of PP2A with its substrates. PP2A has the remarkable ability to interact with structurally distinct regulatory subunits and form complexes with many different substrates owing to the inherent flexibility of the scaffold subunit A, which is composed of 15 tandem HEAT repeats. These 60 holoenzymes catalyze distinct dephosphorylation events that result in specific functional outcomes [79]. PP2A complexes have been implicated in regulation of the mitogen-activated protein kinase, Wnt, PI3K, nuclear factor-**κ**B, protein kinase C, and Ca^2+^/calmodulin-dependent signaling pathways as well as downstream targets of these and other pathways. In most pathways, the specific constituents of the regulatory PP2A complexes have yet to be determined. PP2A dephosphorylates multiple components of these signaling pathways in vitro, and increasing in vivo evidence supports the physiological relevance of many of these interactions [80].

### 5.2. PP2A and Cancer

**Figure 1:**
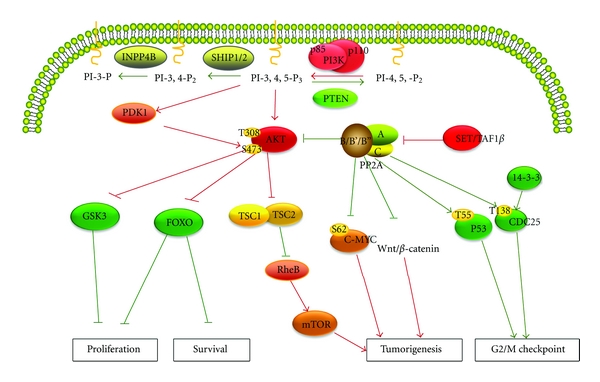
The primary phosphatases function as tumor suppressors and their signaling pathways. This model demonstrates the roles of PTEN, INPP4B, SHIP1/2, and PP2A in regulation of signaling downstream of PI3K/Akt. Two major phospholipid pools—PI(3,4,5)P3 and PI(3,4)P2—were generated in response to stimulation of PI3K. PTEN hydrolyzed the 3′-phosphate of PI(3,4,5)P3 to terminate PI3K signaling. SHIP family members hydrolyzed the 5′-phosphate of PI(3,4,5)P3 to generate PI(3,4)P2, which, like PI(3,4,5)P3, can facilitate PDK1-dependent phosphorylation and activation of AKT. INPP4B converted PI(3,4)P2 to PI(3)P. PP2A not only dephosphorylated Akt at T308 and S473 and negatively regulated the PI3K/Akt pathway but also stabilized p53 or CDC25 and the 14-3-3 complex, inactivated the oncoprotein c-Myc, and antagonized the Wnt/*β*-catenin pathway. Red arrows indicate enhancing tumorigenesis activities, and green arrows indicate inhibition of tumorigenesis.

The role of the tumor suppressor PP2A in controlling tumor progression is thought to be governed by a small subset of specific B subunits directing PP2A to dephosphorylate and regulate key tumor suppressors or oncogenes [76, 81]. Indeed, several members of the B56 family have been described as having a role in directing PP2A's tumor-suppressive activity. PP2A was initially identified as a tumor suppressor in studies in which okadaic acid was found to be a potent carcinoma inducer in a mouse model (Figure 1) [82]. Okadaic acid was also found to be selective inhibitor of PP2A activity in these studies. Ito and colleagues observed that N-terminally truncated B56*γ* leads to enhanced invasiveness and neoplastic progression, transforming melanoma cells from a nonmetastatic to a metastatic state [83]. Further evidence supporting PP2A as a tumor suppressor comes from the finding that the small-t antigen (ST) in two transforming DNA viruses, SV40 and polyoma virus, causes cell transformation by binding to regulatory subunits A and C of PP2A and displacing a single PP2A regulatory subunit (B56*γ*) from PP2A complexes. This interaction is essential for ST to transform cells [84, 85]. Another study confirmed PP2A to be the target of the adenoviral protein E4orf4. It further suggested that PP2A, like other targets of viral oncoproteins, plays an important role in tumor suppression [86]. Mechanistically, downregulation of PP2A expression by ST stabilizes the phosphorylation of proteins such as c-Myc at Ser62 and p53 at either Thr55 or Ser37 and causes cells to undergo uncontrolled growth [87–89]. Chen and colleagues found that specific suppression of the B56*γ* subunit replaced ST of SV40 or polyoma virus and induced cell anchorage-independent growth and tumor formation [87]. The B′/B56/PR61*γ* subunit of PP2A is involved in tumor formation. In addition, partial knockdown of expression of the PP2A*α* subunit results in selective loss of PP2A heterotrimers containing the B56*γ* subunit, and loss of B56*γ* from PP2A complexes substitutes for the small tumor antigen during transformation, as well. The partial suppression of endogenous A*α* leads to activation of Akt kinase, suggesting that activation of the PI3K/Akt pathway contributes to transformation. In addition, PP2A is involved in cell transformation as an important tumor suppressor [79]. Loss-of-function screening on PP2A by short hairpin RNA recognized that PP2A C*α* involved in the SV40 small T-antigen caused human cell transformation but not C*β* subunits or the PP2A regulatory subunits B56*α*, B56*δ*, and PR72/PR130. Further evidence of PP2A as tumor suppressor comes from the finding that inhibition of PP2A expression by short hairpin RNA activates the PI3K/Akt and c-Myc signaling pathways [90].

Although mutations of PP2A A*α* occur at low frequencies in human tumors, mutations of the second PP2A A subunit, A*β*, are more common. Specifically, researchers found somatic alterations, including point mutations, deletions, frameshifts, and splicing abnormalities, of the *PPP2R1B* gene, which encodes the PR65/A scaffold protein, in 15% of primary lung tumors, 6% of lung tumor-derived cell lines, 13% of breast tumors, and 15% of primary colon tumors. Missense mutations and homozygous deletions of the same gene were found in 8% of patients and 2% of patients, respectively, with colorectal cancer [91–94]. These cancer-associated PP2A A*β* mutants are defective in binding to B and/or C subunits in vitro [95]. In addition to mutations of it, the PP2A A*β* gene is located at 11q23, a chromosomal region frequently deleted in cancer cells [96]. Also, PPP2R1A encoding the *α*-isoform of the scaffolding subunit of the serine/threonine PP2A holoenzyme was recently found to be mutated in 7% (3/42) of patients with ovarian clear cell carcinoma [97]. Somatic missense mutations of PPP2R1A have been demonstrated in 41% (20/49) of high-grade serous endometrial tumors and 5% (3/60) of endometrial endometrioid carcinomas. Another study identified mutations of PPP2R1A in ovarian tumors but at lower frequencies: 12% of endometrioid carcinomas and 4% of clear cell carcinomas [98]. Very recently, the PPP2R5E gene, which encodes a regulatory subunit of PP2A, was identified as harboring genetic variants that affect soft tissue sarcoma [99].

### 5.3. PP2A as a Tumor Suppressor

Researchers found that PPP2R1A and PPP2R5E mutations interfered with the binding of specific third regulatory B subunits of PP2A [95]. For example, Damuni's group identified SET as one of the heat-stable PP2A protein inhibitors that induce leukemogenesis. SET, also called template-activating factor 1*β* or phosphatase 2A inhibitor 2, is a nuclear phosphoprotein. SET was first identified in a patient with acute nonlymphocytic myeloid leukemia [100]. The *SET* gene is fused to *CAN * [101]. SET expression is high in rapidly dividing cells but low in quiescent and contact-inhibited cells. SET contributes to tumorigenesis in part by forming an inhibitory protein complex with PP2A [100]. Amino acid residues affected by these mutations are highly conserved across species and interact directly with regulatory B subunits of the PP2A holoenzyme. Additionally, investigators found the B56*γ* mutation F395C, which is located in the B56*γ*-p53 binding domain, in lung cancer cells. This mutation impairs the functions of B56*γ*-PP2A in dephosphorylation of p53 at Thr55 [102].

Furthermore, B56*ε* (encoded by PPP2R5E), a B56-family-regulatory subunit of PP2A, can trigger p53-dependent apoptosis. Mechanistically, B56*ε* regulates the p53-dependent apoptotic pathway solely by controlling the stability of the p53 protein [103].

PP2A reportedly antagonizes the Wnt/*β*-catenin pathway via physical interaction of B56 subunits with Wnt pathway components. In addition, treatment of HEK 293 cells with okadaic acid, an inhibitor of PP2A, results in elevated *β*-catenin protein expression [104]. Overexpression of *PP2A: B56*ε** inhibits Wnt/*β*-catenin signaling in tissue culture and *Xenopus* embryos [104–106]. Loss-of-function analysis of PP2A: B56*ε* during early *Xenopus* embryogenesis showed that PP2A: B56*ε* is required for Wnt/*β*-catenin signaling [107]. The B′/B56/PR61 subunit binds to the tumor suppressor adenomatous polyposis coli, which is a component of the Wnt pathway. The Wnt pathway plays essential roles during embryonic development and tumorigenesis [108, 109]. B56*α*-PP2A can dephosphorylate c-Myc at Ser62 and inactivate the oncoprotein c-Myc [110, 111]. The protein cancerous inhibitor of PP2A interacts directly with the oncogenic transcription factor c-Myc by inhibiting the catalytic activity of the PP2A holoenzyme toward c-Myc at Ser62, thereby preventing c-Myc proteolytic degradation without affecting PP2A binding potential [112].

PP2A is involved in regulation of DNA-responsive G2/M checkpoints, as well. DNA-responsive checkpoints activate PP2A/B56*δ* phosphatase complexes to dephosphorylate CDC25 at sites different from Ser287 (Thr138), phosphorylation of which is required for release of 14-3-3 protein from CDC25. Ser287 phosphorylation is a major locus of G2/M checkpoint control. B56*δ* C-PP2A promotes Thr138 dephosphorylation and prevents 14-3-3 release. This restricts PP1 recruitment, CDC25 activation, and entry of cells from G2 to M phase. Remarkably, the CHK1 kinase activated during the replication checkpoint phosphorylates B56*δ*, enhancing its incorporation into PP2A holoenzyme. Therefore, B56*δ*-PP2A dephosphorylates Cdc25, blocking cell-cycle progression as a central checkpoint effector [113, 114]. However, whether PP2A/B56*δ* phosphatase complexes are involved in DNA repair must be clarified.

In other experiments, researchers identified B56-containing PP2As to be phosphatases of Akt and found that PP2A reverses immediate early response gene X-1–mediated Akt activation [115]. Immediate early response gene X-1, also known as *IER3*,* DIF2*, and *Gly96*, is an ubiquitous early response gene product involved in cell proliferation and survival. The cell proliferation and survival is rapidly induced in response to various growth factors, cytokines, chemical carcinogens, and viral infections [116]. Vereshchagina and colleagues found that the protein phosphatase PP2A-B′ subunit Widerborst acts as a subcellular compartment-specific regulator of PI3K/PTEN/Akt kinase signalling and negatively regulates cytoplasmic Akt activity in *Drosophila* [117]. A more recent study confirmed that B56*β* (PPP2R5B, B′*β*) plays a critical role in the assembly of the PP2A holoenzyme complex on Akt, which leads to dephosphorylation of both Ser473 and Thr308 Akt sites. However, Cdc2-like kinase 2 phosphorylates the PP2A regulatory subunit B56 *β* and triggers the assembly procession of PP2A holoenzyme complex and subsequently downregulates Akt activity [118]. Moreover, a study identified PP2A (encoded by PPP2R5E) along with BIM (Bcl2L11), an AMP-activated kinase (encoded by *Prkaa1*), and the tumor suppressor phosphatase PTEN as the targets of miRNA-19 in Notch-induced acute T-cell leukemia cells [119]. 

In general, the genetic and epigenetic changes in PP2A complexes in human cancer cells remain to be defined, as does their impact on cancer signaling and therapeutic responses to targeted therapy. One of the PP2A-regulated cancer signaling pathways is the mammalian target of rapamycin pathway, a key component of the PI3K pathway that many cancer cells are “addicted” for growth.

## 6. Conclusion

SHIP1/2, PP2A, INPP4B, and PTEN are commonly viewed as opposing the activity of the PI3K/Akt signaling axis, which promotes survival of cancer cells and tumors. It is certain that the enzymatic activities of 3′ polyphosphatase work as negative controller. Most powerfully, PTEN downregulates PI3K's reaction by converting PI(3,4,5)P_3_ to PI(4,5)P_2_. Whereas the 5′ polyphosphatase activity of SHIP1/2 converts PI(3,4,5)P_3_ to PI(3,4)P2. This distinction is potentially crucial, as it may enable SHIP1/2 and PTEN to have distinctly different effects on Akt signaling. PTEN expression is a relatively ubiquitous negative regulator of the PI3K/Akt signaling pathway. Loss-of-function PTEN mutation/deletions lead to the development of all types of cancer. SHIP1 is specifically expressed in all cells of the hematopoietic system and is correlated with T- and B-cell lymphoma development. SHIP2 functions as a positive regulator of the epidermal growth factor receptor/Akt pathway, C-X-C chemokine receptor type 4 expression, and cell migration in breast cancer cells but a negative regulator of keratinocyte migration. INPP4B specifically hydrolyzes PI(3,4)P2 to be PI(3)P, negatively regulates the PI3K/Akt pathway, and has emerged as a potential tumor suppressor in prostate, breast, and ovarian cancers and, possibly, leukaemias. PP2A, as a tumor suppressor, is more complicated than other phosphatases because it has five regulatory subunits that exist in different tissues and play different roles in various cells. These five subunits are inclined to be mutated and affect their own function. Most of the mutations of these five subunits remain unidentified. How PTEN, SHIP1/2, INPP4B, and PP2A orchestrate to sustain normal signaling and achieve efficient inhibition of the PI3K/Akt pathway in all types of cells and tissues is still far from being completely determined.

##  Conflict of Interests

The authors declared that there is no conflict of interests.
